# ANP32A dysregulation contributes to abnormal megakaryopoiesis in acute megakaryoblastic leukemia

**DOI:** 10.1038/s41408-017-0031-x

**Published:** 2017-12-22

**Authors:** Xueqin Sun, Bin Lu, Cuijuan Han, Wanlin Qiu, Qi Jin, Dengju Li, Qiubai Li, Qiong Yang, Qiang Wen, Puneet Opal, Ameet R. Kini, John D. Crispino, Zan Huang

**Affiliations:** 1 College of Life Sciences, Hubei Key Laboratory of Cell Homeostasis, Wuhan University, Wuhan, China; 2 Department of Hematology, Tongji Hospital of Tongji Medical College, Huazhong University of Science and Technology, Wuhan, China; 3 Institute of Hematology, Union Hospital of Tongji Medical College, Huazhong University of Science and Technology, Wuhan, China; 4 Feinberg School of Medicine, Northwestern University, Chicago, IL USA; 5 Chicago Stritch School of Medicine, Loyola University, Chicago, IL USA

Acute megakaryoblastic leukemia (AMKL) is a rare type of leukemia characterized by indefinite proliferation of megakaryocytes^1^. The prognosis of AMKL is dismay and no target therapy is available that urges for development of novel therapy^2^. Recent research proposed that forcing AMKL cells to undergo polyploidization and differentiation was a good therapeutic strategy for AMKL^3^. Thus, regulators controlling megakaryopoiesis could be potential targets for AMKL therapy. *ANP32A* gene was implied to be a potential regulator of hematopoiesis and megakaryopoiesis^4^. However, its role in blood remains unclear.

In this study, we observed a potential correlation between ANP32A downregulation and megakaryocyte differentiation. Hematopoietic stem cells (HSCs) (CD133^+^CD34^dim^) and megakaryocyte-erythrocyte progenitor expressed a higher level of ANP32A than colony-forming unit-megakaryocyte (CFU-Mk) and mature megakaryocytes (Fig. 1a)^5^, and significant upregulation of ANP32A was verified in primary AMKL cells (Fig. 1b). However, ANP32A was downregulated in leukemic cells undergoing megakaryocytic differentiation (Supplementary Fig. 1A, B). Interestingly, complete blood count of *Anp32A* ^*−/−*^ mice were apparently normal (data not shown). Both *Anp32A* ^*−/−*^ and ANP32-overexpressing megakaryocyte cultures showed comparable CD41 and CD42 expression compared with wild-type (WT) cells. ANP32A-deficient megakaryocytes only exhibited mild increased of polyploidy in CD42^+^ megakaryocytes and slight decrease of CFU-Mk, whereas ANP32A overexpression had an opposite but marginal effect (Supplementary Fig. 2A–F). These observations suggest a dispensable role of ANP32A on normal megakaryopoiesis. This may be due to the compensatory effect of *ANP32B* and *ANP32E* as proposed previously^6^. In sharp contrast, ANP32A knockdown (shANP32A#1) in 6133/MPL W515L cells induced spontaneous megakaryocytic differentiation in the absence of phorbol 12-myristate 13-acetate (PMA) with increased CD41 and CD42 expression (Fig. 1c), which was confirmed in multiple AMKL cell lines (Supplementary Fig. 3A–F). Although ANP32A overexpression failed to promote K562 cell proliferation, it did impair PMA-induced megakaryocytic differentiation (Supplementary Fig. 4A–C). Furthermore, ANP32A knockdown significantly reduced colony-forming ability of these cells in soft agar (Fig. 1d). Notably, ANP32A downregulation significantly impaired the ability of 6133/MPL W515L cells to induce AMKL in mice^7^ and improved the survival rate (Fig. 1e). These observations indicate that ANP32A may be critical for AMKL cell to maintain hyper-proliferative and undifferentiated status and contribute to the pathogenesis of AMKL.

**Fig. 1:**
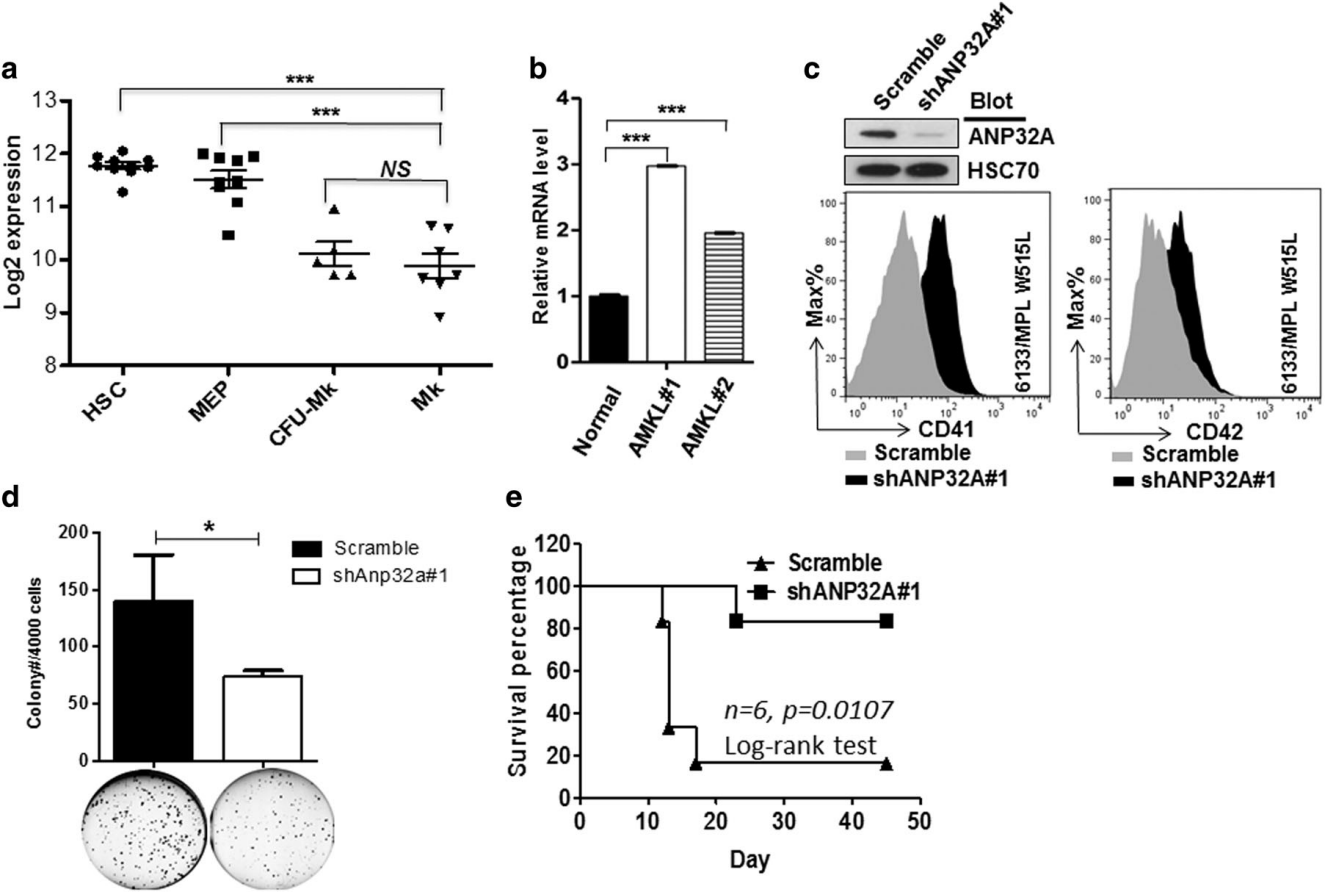
ANP32A dysregulation contributes to AMKL **a** The expression of ANP32A mRNA in HSC CD133^+^CD34^dim^, MEP (megakaryocyte-erythrocyte progenitor), CFU-Mk (colony-forming unit-megakaryocyte), and Mk (megakaryocytes) were analyzed and presented as log 2 expression. Expression data were obtained from online Bloodspot database (http://servers.binf.ku.dk/bloodspot/?gene=C5orf4&dataset=DMAP). **b** Quantitative RT-PCR analysis of *ANP32A* in MNCs from healthy donors (Normal, *N*=5) and two cases of AMKL patients (AMKL#1 and #2). The expression of ANP32A was normalized to GAPDH and presented as relative mRNA level. ***p < 0.001; NS: not significant. **c** Immunoblotting to detect ANP32A expression and flow cytometry to measure the expression of CD41 and CD42. Histograms were representative results of three independent experiments (duplicates) with similar results. **d** Scramble or Anp32a-knockdown (shAnp32a#1) 6133/MPL W515L cells were seeded in soft agar to measure the CFU. *p < 0.05. **e** Scramble or ANP32A-knockdown 6133/MPL W515L cells (shANP32A#1) were transplanted into semi-lethally irradiated mice through retro-orbital injection. The mice survival was observed up to 7 weeks

Mechanistically, ectopic expression of ANP32A dampened the induction of RUNX1 and FLI1 and inhibited extracellular-signal-regulated kinase (ERK) activation by phorbol myristate acetate (PMA) (Fig. 2a). In contrast, ANP32A downregulation caused an opposite phenotype (Fig. 2b). These findings were consistent to previous reports showing that PMA induces activation of mitogen-activated protein kinase/ERK and stress-activated protein kinase/c-Jun NH(2)-terminal kinase pathways and subsequently regulate the expression of RUNX1 and FLI1 to promote megakaryopoiesis^8, 9^. Noticeably, further RUNX1 knockdown (shANP32A#3+shRUNX1) or FLI1 knockdown (shANP32A#3+shFLI1) efficiently abrogated shANP32A#3-induced megakaryocytic differentiation (Fig. 2c, Supplementary Fig. 5). Moreover, ERK inhibitor PD98059 significantly suppressed the induction of RUNX1 and FLI1 expression and abolished shANP32A#3-induced megakaryocytic differentiation (Fig. 2d, e). Interestingly, ANP32A knockdown in primary AML cells increased the expression of RUNX1 and FLI1 and enhanced ERK phosphorylation while GATA1 was intact (Fig. 2f). Our findings suggest that ANP32A may inhibit ERK and subsequently repress RUNX1 and FLI1 to promote megakaryocyte differentiation.

**Fig. 2:**
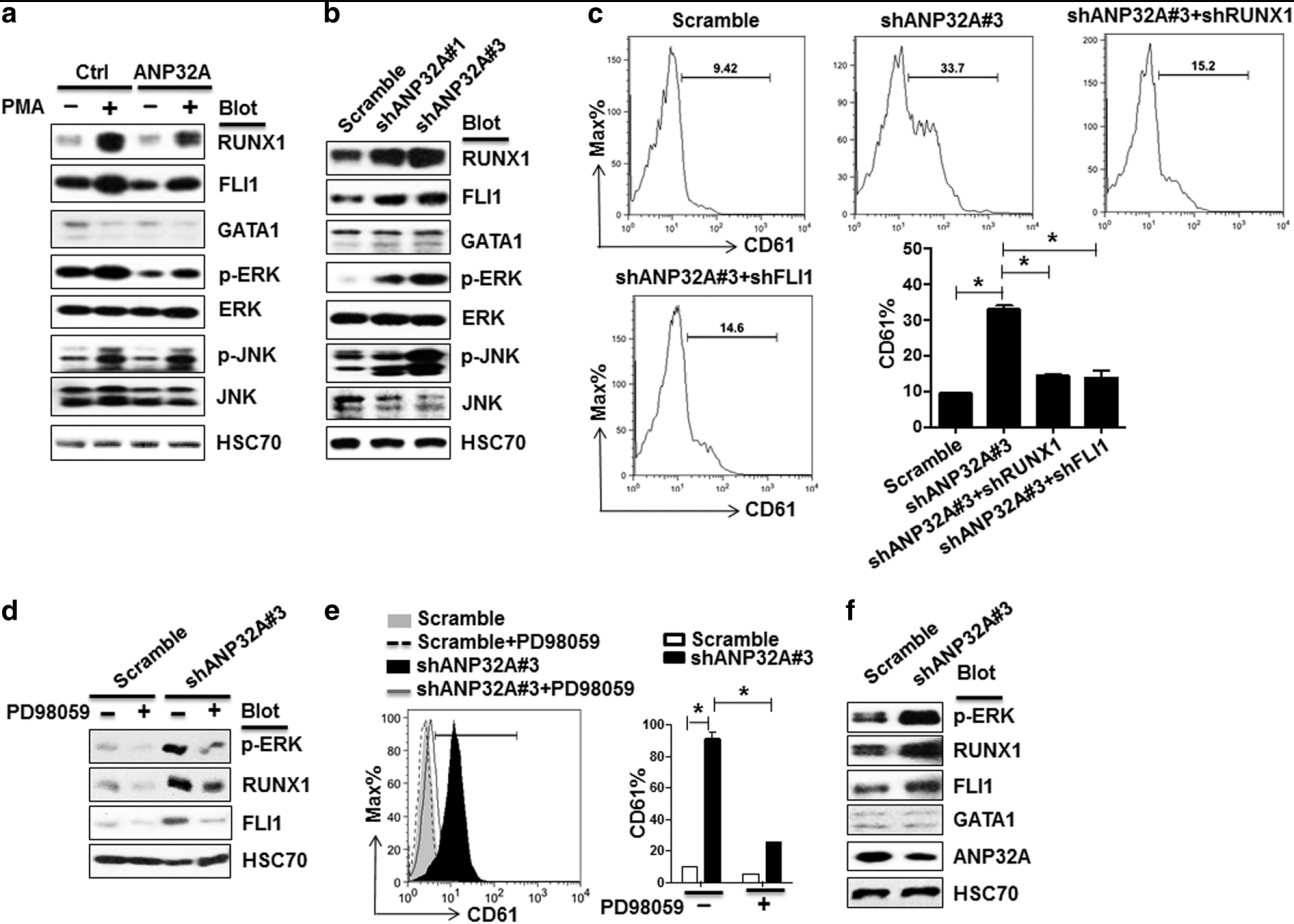
ANP32A impairs megakaryocyte differentiation by repressing ERK signaling and subsequent RUNX and FLI1 expression **a** Immunoblotting to detect protein expression and phosphorylation in control and ANP32A-expressing K562 cells treated with (+) or without (−) PMA for 2 days. HSC70 served as a loading control. **b** Immunoblotting to detect protein expression and phosphorylation in Scramble or ANP32A-knockdown K562 cells (shANP32A#1, shANP32A#3). HSC70 served as a loading control. **c** RUNX1 or FLI1 was further knocked down in ANP32A-knockdown K562 cells. CD61 expression in the resultant cells was measured by flow cytometry. Histograms were representative results of three independent experiments (duplicates) with similar results. *p < 0.05. **d** Immunoblotting to detect RUNX1, FLI1 expression, and ERK phosphorylation in Scramble or ANP32A-knockdown (shANP32A#3) K562 cells treated with or without PD98059. HSC70 served as a loading control. **e** Flow cytometry to measure CD61 expression in the resultant cells in **d**. Histogram was representative data from three independent experiments (duplicates) with similar results. *p < 0.05. **d** Immunoblotting to detect protein expression and phosphorylation in Scramble or ANP32A-knockdown primary AML cells (shANP32A#3). HSC70 served as a loading control

In summary, our study reveals that ANP32A dysregulation may be a critical factor contributing to AMKL and ANP32A may be a good target for AMKL therapy. Previous studies showed that ANP32A bound to unmodified histone H3 and inhibited H3 acetylation^7^. Thus, ANP32A downregulation may potentially alter global epigenetic modifications.

## Electronic supplementary material

supplementary information and data
