# Supplementary material for: ANP32A dysregulation contributes to abnormal megakaryopoiesis in acute megakaryoblastic leukemia
Source: Blood Cancer J. 2017 Dec 22;7(12):661. doi: 10.1038/s41408-017-0031-x (PMC5802576; doi:10.1038/s41408-017-0031-x)
Supplement: Supplementary file 1 — supplementary information and data [file 41408_2017_31_MOESM1_ESM.docx]

**Materials and methods**

**Cell cultures, animals and blood samples**

Leukemic cell lines K562, HEL, CMK, CHRF, Meg-01, 6133/MPL W515L were cultured in RPMI 1640 medium (Gibco BRL, Grand Island, NY, USA) and HEK293T cells were cultured in Dulbecco’s modiﬁed Eagle medium (DMEM) (Gibco BRL, Grand Island, NY, USA) supplemented with 10% fetal bovine serum, streptomycin, and penicillin, at 37°C under 5% CO2. Set2 cells were maintained in RPMI 1640 medium with 20% FBS. *Anp32a^-/-^* and wild-type (C57BL) mice were maintained in a barrier mouse facility. All animal studies were approved by the Animal Care and Use Committees of College of Life Sciences of Wuhan University and Feinberg School of Medicine of Northwestern University. Mononuclear cells (MNCs) were isolated from peripheral blood samples on Ficoll-Hypaque density gradients. All experiments involving in human blood samples were approved by the Medical Ethics Committees of Tongji Hospital of Tongji Medical College and Union Hospital of Tongji Medical College, Huazhong University of Science and Technology. Consent form was obtained from each patient and healthy donor.

**Megakaryocyte culture and colony-forming unit megakaryocytes**

Megakaryocyte culture and colony-forming unit megakaryocytes were performed as previously described. 5×10^4^ lineage-negative (lin^-^) bone marrow cells were seeded in Megacult-C medium for the analysis of pure megakaryocyte colonies (CFU-MK).

**Lentivirus or retrovirus infection**

Overexpression and knockdown cell lines were obtained by lentiviral or retroviral transduction as previously described. Lentiviral vector pHAGE or retroviral vector pMSCVpuro was used for overexpression and lentiviral vector pLKO.1 was used for knockdown. Vectors carried puromycin resistant gene or GFP for selection as indicated. Empty vectors were used as negative controls. Short hairpin RNA sequences for ANP32Aknockdown were determined by online shRNA searching tool (http://www.sirnawizard.com/design_advanced.php), and blasted at NCBI to avoid off-target effect. The shRNA sequences specific for human *ANP32A* used in this study were as following: shANP32A#1: CCTATTGTGATTTGACTGTTT, shANP32A#3: CCTGAAGATGAGGGAGAAGAT. The shANP32A#3 targets the UTR region of *ANP32A* mRNA that is identical to that of mouse *Anp32a* mRNA. Therefore, we used shANP32A#3 to downregulate *Anp32a* in mouse cell lines. The shRNA sequence for RUNX1 knockdown were shRUNX1: GGCACTCTGGTCACCGTCA. The shRNA sequence for FLI1 knockdown were: shFLI1: GGCACAAACGATCAGTAAGA. The vector pLKO.1-scramble shRNA containing scrambled shRNA were used as a negative control.

**Flow cytometry to analyze megakaryocytic differentiation**

Cells were washed with PBS and stained with phycoerythrin (PE)-conjugated anti-CD61 antibody, allophycocyanin (APC)-conjugated anti-CD41 antibody, or PE-conjugated anti-CD42 antibody (BD Biosciences, San Jose, CA, USA). CD61, CD41, or CD42 expression was measured by flow cytometry on FACSCalibur (BD Biosciences, San Jose, CA, USA) and analyzed with FlowJo software (TreeStar, Ashland, OR). All flow cytometry data were the representative results from three independent experiments with duplicates.

**Quantitative RT-PCR Analysis**

Total RNA was extracted from cells using TRIzol reagent (Invitrogen, Grand Island, NY, USA), and reverse-transcribed into cDNA using High-Capacity Reverse Transcription kit (Applied Biosystems, Grand Island, NY, USA). QRT-PCR was performed as following conditions: 95°C for 15 min followed by 95°C for 30 sec, 60°C for 30 sec, 72°C 1 min for 40 cycles. The reactions were run in duplicate on the 7900HT Fast Real-Time PCR System (Applied Biosystems, Grand Island, NY, USA). The relative quantitation of real-time PCR product was measured using the comparative ΔΔC_T_ method. All quantitative RT-PCR data were the representative results from three independent experiments with duplicates. Sequences of primer sets used for quantitative RT-PCR are available upon requested.

**Immunoblotting and antibodies**

Immunoblotting was performed as previously described. Antibodies used in this study include goat anti-GATA-1, anti-ANP32A, rabbit anti-FLI1, mouse anti-RUNX1, mouse anti-HSC70 (Santa Cruz Biotechnology, Santa Cruz, CA, USA); rabbit anti-ERK, anti-phospho-ERK, anti-JNK, anti-phospho-JNK, anti-phospho-p38, anti-phospho-NF-κB (Cell Signaling Tech); mouse anti-HA (ProteinTech Group, Chicago, IL, USA); mouse anti-Flag(Sigma, St Louis, MO, USA). HSC70 antibody served as loading controls. Goat anti-mouse, donkey anti-goat, and goat anti-rabbit IgG horseradish peroxidase (HRP)-conjugated were purchased from Jackson ImmunoResearch. All　immunoblots were the representative data from three independent experiments with similar results.

**Colony-forming assay**

Briefly, cells were harvested, single cell suspension was prepared, and cell density was determined. An agar (2.0%) underlay was plated in a 35mm cell culture dish. Cells (4×10^3^) were mixed with pre-warmed soft agar suspension (0.3%) and the mixtures were plated on the top of underlay agar. Cells were cultured for up to 3 week and the cell colonies were counted under microscope.

**AMKL mouse model**

AMKL mouse experiment was performed as previously described. Briefly, wild type mice (C57BL) were sub-lethally irradiated (600 rad) and received 6133/MPL W515L cells (0.2×10^6^ cells/mouse) through retro-orbital injection. The engraftment was confirmed by flow cytometry to analyze the GFP+ cells in the peripheral blood of recipient mice 5 days post transplantation. The disease burden and survival in the recipient mice were monitored over time.

**Statistics**

All statistical analyses except survival curve were performed using the Student’s *t* test (2 tailed, unpaired). To compare survival curve, Log-rank (Mantel-Cox) test was used. A *p* value of 0.05 or less was considered significant.





**Supplementary Figure S1. ANP32A downregulation correlates to megakaryocyte differentiation.**

(A) K562 and (B) HEL cells were treated with PMA for days as indicated. The expression of ANP32A was determined by quantitative RT-PCR.





**Supplementary Figure S2. *Anp32A*-deficiency marginally affects megakaryopoiesis.**

(A) Bone marrow progenitor cells from WT and *Anp32A^-/-^* mice were cultured and induced to differentiation into megakaryocyte. The CD41 and CD42 expression were measured by flow cytometry. (B) The polyploidy of the resultant CD42^+^ cells were also analyzed. (C) WT and *Anp32A^-/-^* bone marrow cells were also used for CFU-Mk assay. (D) The WT bone marrow progenitor cells were infected with control (Ctrl) or ANP32A-overexpressing retrovirus and induced to differentiate intro megakaryocytes. The CD41 and CD42 expression were measured. (E) The polyploidy of the resultant CD42+ cells were also analyzed. Data was the statistics from two mice. (F) The transduced bone marrow progenitor cells were also used for CFU-Mk assay. *: *p*<0.05; *NS*: non-significant.





**Supplementary Figure S3. Downregulation of ANP32A triggers spontaneous megakaryocytic differentiation in multiple leukemic cells**

(A) K562, (B)HEL, (C)CMK, (D)CHRF, (E)Meg-01, (F)Set2 cells were transduced with control lentiviral vector (Scramble) or lentiviral vectors expressing shRNAs specific for human ANP32A (shANP32A#1 or shANP32A#3). The endogenous protein levels of ANP32A in resultant cells were determined by western blot with specific antibody against ANP32A. HSC70 served as a loading control. The expression of CD61, CD41, or CD42 in these cells was detected by flow cytometry. Histograms were representative results of three independent experiments (duplicates) with similar results.





**Supplementary Figure S4. ANP32A upregulation appeared to affect abnormal megakaryocyte differentiation in K562 cell.**

(A) Ectopic ANP32A expression was determined by western-blot. (B) Cell population was counted everyday for four days. (C) K562 cells overexpressing ANP32A and empty control were treated with PMA (+) or without (-) for 2 days. CD61 expression was determined by flow cytometry. Histogram was representative data from three independent experiments (duplicates) with similar results. A *p*-value <0.05 was considered as significant.





**Supplementary Figure S5.** **The function of ANP32A knockdown is mediated by RUNX1 and FLI1.**

(A) K562 cells were transduced with control (Scramble), ANP32A-knockdown lentiviral vectors (shANP32A#3), both ANP32A and RUNX1 knockdown (shANP32A#3+shRUNX1) vectors. The expression of ANP32A and RUNX1 was determined by western blot. HSC70 served as a loading control. (B) K562 cells were transduced with control (Scramble), ANP32A-knockdown lentiviral vectors (shANP32A#3), both ANP32A and FLI1 knockdown (shANP32A#3+shFLI1) vectors. The expression of ANP32A and FLI1 was determined by western blot. HSC70 served as a loading control.
